# Subjective hearing loss is not associated with an increased risk of Alzheimer's disease dementia

**DOI:** 10.1016/j.heliyon.2024.e30423

**Published:** 2024-05-03

**Authors:** Meher Lad, John-Paul Taylor, Tim D. Griffiths, for the Alzheimer's Disease Neuroimaging Initiative

**Affiliations:** a Translational and Clinical Research Institute, Newcastle University, Newcastle upon Tyne, United Kingdom; b Biosciences Institute, Newcastle University, Newcastle upon Tyne, United Kingdom; c Wellcome Centre for Human Neuroimaging, University College London, London, United Kingdom

**Keywords:** Alzheimer's disease, Dementia, Hearing loss, Cognition, Neuroimaging

## Abstract

Hearing loss is a risk-factor for dementia but the reasons for this are unclear. Subjective hearing loss is related to increased future dementia risk, however, this metric has not been previously examined with cognitive, neuroimaging and biochemical measures that are relevant to Alzheimer's disease. We assessed Cognitively Normal and Mild Cognitively Impaired participants from the Alzheimer's Disease Neuroimaging Initiative with subjective hearing loss to examine if they had faster decline in episodic memory scores, hippocampal volume and greater pTau positivity. The likelihood of a dementia diagnosis in hearing impaired participants over a 5-year period was also assessed. There were no statistically significant differences between the hearing subgroups over a 5-year period nor were there in conversions to a dementia diagnosis. Objective hearing loss metrics may provide a more reliable link between hearing loss and dementia risk.

## 1 Introduction

Hearing loss in midlife (between the ages of 45 and 60) is associated with an increased risk of dementia in the future [[1], [2], [3]]. Studies that have provided this evidence have usually used the pure-tone audiogram (PTA), a test which objectively measures minimum intensity perceived by an individual for a range of tones, at different frequencies, from 250 Hz to 8 kHz, in silence. These studies have found associations with hearing loss severity; severe hearing loss has the greatest risk of dementia compared moderate hearing loss, mild hearing loss and no hearing difficulty. However, these studies have not measured other hearing abilities that may have associations with PTA scores, such as speech-in-noise (SiN) perception. The latter has also been associated with dementia risk [4,5]. People with severe SiN perception impairment are also at greater risk of dementia than people with mild impairments and those with no impairments.

Prior work has suggested that objective hearing measures are specifically linked to risk of Alzheimer's disease (AD) dementia, potentially through a neurobiological process. The hearing loss could therefore be an indicator of the AD disease process itself. Poor hearing on the PTA is associated with faster rates of medial temporal lobe atrophy over time, a brain region that is affected early with neuropathology in AD [6]. Poor SiN hearing metrics are also linked cross-sectionally to higher cerebrospinal fluid (CSF) phosphorylated-Tau-181 (pTau-181) levels, a sensitive biochemical marker that predicts conversion to AD dementia [5]. pTau-181 levels capture abnormal Tau accumulation in response to amyloid pathology [7].

Although objective hearing measures provide robust and reliable values for an individual, there are disadvantages to using these tests, which may be overcome by using subjective measures. Objective tests are more time-consuming than subjective tests and require specialist equipment and training. They may not accurately reflect an individual's everyday listening experience, as the hearing tests are typically performed under controlled laboratory conditions. Real-world listening environments can be much more complex and dynamic than those encountered in a soundproof booth, making it challenging to accurately assess an individual's hearing abilities. Subjective hearing loss is easier to measure and has been associated with objective hearing loss by PTA thresholds [8]. A single question about hearing loss, “Do you feel you have hearing loss?” has greater than 90 % sensitivity for PTA defined moderate to severe hearing loss in some work [9]. If a relationship between any hearing loss measure and dementia risk exists, then it could be argued that subjective hearing loss could identify people with the greatest risk of future dementia.

Some studies that have used subjective measures of hearing loss by direct questioning have also elicited a dementia risk. One study found poor subjective hearing is linked to poor episodic memory in the form of immediate and delayed recall for word lists [10]. Clinician-judged hearing impairment has also been linked with more severe AD pathology in cognitively normal individuals and with a greater burden of vascular and Lewy body pathology post-mortem [11]. Therefore, it is plausible that subjective hearing loss may also capture the relationship between hearing loss and dementia via the same mechanisms as objective measures.

There have been mixed findings of associations between subjective hearing loss and AD dementia. Self-reported hearing impairment has been associated with an increased risk of Mild Cognitive Impairment (MCI), a known precursor to dementia [12]. Subjective hearing difficulties during non-auditory clinical assessments have also been associated with an increased rate of developing dementia and cognitive decline [13]. However, another study found no change in any known neurodegenerative disease in post-mortem brain samples of those with and without subjective hearing loss [14]. Further work is necessary to understand the factors that underlie these findings such as whether subjective hearing loss predicts clinical markers of dementia *in-vivo* as post-mortem studies may not adequately capture an temporal relationship between the time hearing loss was measured and the onset of dementia.

In this study, we sought to test whether subjective hearing loss increases the risk of AD dementia by considering neuropsychological, neuroimaging and biochemical markers that are specifically linked to AD dementia diagnosis or its risk in the future [[15], [16], [17]]. We examined whether stable cognitively normal and MCI individuals with subjective hearing loss had greater changes in episodic memory scores, hippocampal volumes, and pTau-181 CSF levels over time in the Alzheimer's Disease Neuroimaging Initiative (ADNI) cohort. The benefit of studying this population is twofold: 1) there are a larger number of participants whose clinical diagnosis stays unchanged over a 5-year period and this gives more power to identify meaningful changes in biomarkers, 2) some individuals may develop AD dementia over much larger time scales and the first indication of an increased risk of dementia may be a deviation from a healthy population by showing faster changes in cognitive, neuroimaging or fluid biomarker metrics.

Furthermore, we also studied these variables in those participants with MCI who converted to AD dementia over a 5-year period to study if hearing loss was associated with faster episodic memory decline, hippocampal volume loss and changes in phosphorylated Tau CSF levels. This would support subjective hearing loss as a metric to predict the onset of dementia. Studying this population is crucial as it provides a direct evaluation of people in the preclinical stages of dementia. Therefore, any increase in risk for dementia due to subjective hearing status can be directly attributed to this variable.

## 2 Methods

### 2.1 The Alzheimer's disease neuroimaging initiative

Data used in the preparation of this article were obtained from the ADNI database (adni.loni.usc.edu). The ADNI was launched in 2003 as a public-private partnership, led by Principal Investigator Michael W. Weiner, MD. The primary goal of ADNI has been to test whether serial magnetic resonance imaging (MRI), positron emission tomography (PET), other biological markers, and clinical and neuropsychological assessment can be combined to measure the progression of mild cognitive impairment (MCI) and early Alzheimer's disease (AD). For up-to-date information, see www.adni-info.org.

### 2.2 Participants

**Fig. 1:**
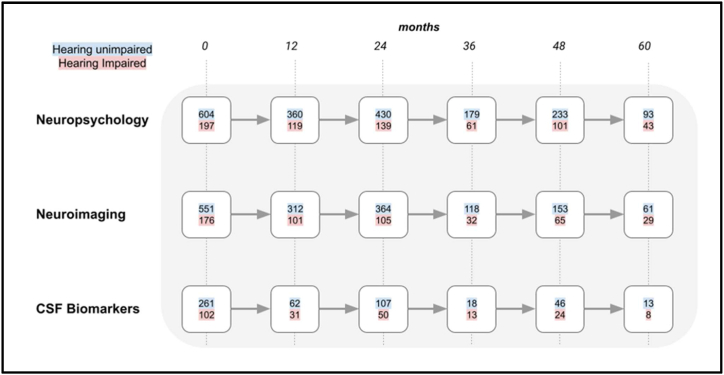
A flowchart showing the number of cognitively normal participants that remained with that diagnosis at 12-month intervals. The different rows indicate the total number of participants that completed neuropsychological, neuroimaging or CSF biomarker evaluation. The number of hearing unimpaired participants are indicated with a blue background and those with self-reported hearing impairment have a red background. (For interpretation of the references to colour in this figure legend, the reader is referred to the Web version of this article.)

**Fig. 2:**
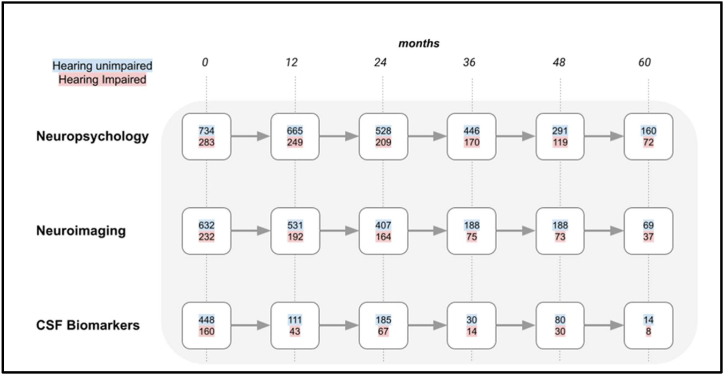
A flowchart showing the number of participants with MCI remained with that diagnosis at 12-month intervals. The different rows indicate the total number of participants that completed neuropsychological, neuroimaging or CSF biomarker evaluation. The number of hearing unimpaired participants are indicated with a blue background and those with self-reported hearing impairment have a red background. (For interpretation of the references to colour in this figure legend, the reader is referred to the Web version of this article.)

The ADNI database (http://adni.loni.usc.edu) has coded participant data that can be accessed for analysis with prior authorisation. As this study examined hearing loss as a predictor of AD dementia, only data from people without a diagnosis of AD at baseline was used for analysis. Therefore, people who were cognitively normal (including participants with subjective memory complaints), and MCI were included in the study. Their data from 12-, 24-, 36-, 48- and 60-month follow-ups were used for analyses. Participants with MCI who converted to a diagnosis of AD dementia at 12, 24, 36, 48 and 60 months became part of a group that were analysed separately to assess the predictive value of subjective hearing loss for subsequent dementia. These are depicted in flowcharts in Fig. 1, Fig. 2. When AD dementia developed in the follow-up period, it was defined in the study as per the NIA-AA consensus criteria [18]. Further details about the inclusion criteria for the study can be found on the ADNI webpage (http://adni-info.org).

People with subjective hearing impairment were identified by using two methods. The first was through the baseline physical examination in which participants declared if they had hearing loss (of any severity) or not. There was no structured interview or question used to determine this. Only the presence of hearing loss was recorded. This was determined by the clinician during interaction with a participant or self-report. Hearing loss was coded as present if this was present at every visit. The second method included the examination of ‘free-text’ records including a participant's past medical history, active problems or miscellaneous information. As objective data on hearing measurements was not available, all mentions of degrees of hearing loss (mild, moderate and severe) and laterality were combined into one variable that indicated whether hearing loss was present or absent. Both these methods have been used in a similar way previously to identify people with hearing loss in the ADNI dataset [19]. Although there is a substantial overlap in the participants identified by each method, a combination of these increases the yield of identifying people with ‘hearing loss’ in a binary fashion by around 20 %. No mention of any hearing problems was coded as hearing loss not being present. A single variable that coded whether a participant had reported hearing loss or not was the end result. Due to the paucity of reliable and objective data on hearing aids and their usage, this metric was not used for further analysis in this study. Participants with cochlear implants were not recruited to this study as this was part of the exclusion criteria. There was no available information on balance disorders collected in a systematic way, which is a common finding with people with sensorineural hearing loss and could affect the results.

### 2.3 Neuropsychological assessment

The Rey Auditory Verbal Learning Test (RAVLT) is a widely used neuropsychological test that assesses various aspects of memory, including immediate recall, delayed recall, and recognition. The first two measures were combined and used as a total composite score for further analysis in this study. Immediate recall is the first component of the test and involves the participant's ability to immediately recall a list of 15 unrelated words that are presented orally by the examiner [20]. The procedure for immediate recall component of the RAVLT involves the following steps: the examiner reads a list of 15 unrelated words, the examiner immediately asks the participant to recall as many words as they can remember, The examiner repeats the same list of 15 words a total of five times, with the participant being asked to recall the words after each presentation and After the fifth presentation, the examiner asks the participant to recall as many words as they can remember from any of the five presentations.

The Mini-Mental State Examination (MMSE) is a widely used screening tool for assessing cognitive impairment and dementia. It is a brief, standardised test that assesses various cognitive domains, including orientation, memory, attention, language, and visuospatial skills. The test consists of 30 questions and takes approximately 10 min to complete. It is scored out of a maximum of 30 points, with a score of 24 or higher indicating normal cognitive functioning. The MMSE is a useful tool for identifying cognitive impairment and monitoring changes in cognitive status over time, and was used as a global measure of cognition.

The RAVLT and MMSE both have components that rely on verbal instructions and presentation of stimuli to remember and so it is possible that poor hearing may potentially result in an artificially low performance for hearing-impaired participants. However, these tests were chosen as they are commonly administered neuropsychological tests at all timepoints for all participants and are common measures in dementia studies.

A participant had measurements for the RAVLT and MMSE test at each time point. The change in RAVLT and MMSE scores, but not absolute values, at each time point was used as the variable of interest for further analysis.

### 2.4 Neuroimaging assessment

Cortical reconstruction and volumetric segmentation of MRI scans from the ADNI dataset were performed with the FreeSurfer 6.0 image analysis pipeline, which is a widely-used tool for automatic brain imaging analysis. It is designed to identify and label various cortical and subcortical structures, such as the grey matter, white matter, cerebrospinal fluid, and different gyri and sulci of the brain. FreeSurfer utilises advanced image processing algorithms, including intensity normalisation, tissue classification and surface deformation, to generate accurate and reliable 3D models of the brain. For the ADNI study, volumes of various Regions of Interest (ROI) used in this study were available in a tabulated format from the study website after being processed from each study site. Whole brain and hippocampal volumes for both cerebral hemispheres were combined and normalised by a participant's total intracranial volume to produce a metric each for whole brain volume and hippocampal volume. These values were used for further analysis. Changes in these values over time for an individual were used for further analysis.

### 2.5 Biochemical assessment

A subgroup of ADNI participants underwent a lumbar puncture to obtain CSF samples for biomarker analysis. pTau-181 levels were analysed at the University of Pennsylvania using the Roche Elecsys in vitro diagnostic immunoassay intended for quantitative determination of protein levels in pg/mL. Previous work has identified that a pTau-181 value of above 38.2 pg/mL has a high sensitivity and sensitivity for predicting conversion to AD dementia from MCI [21]. This limit was used to determine pTau positivity in an individual.

### 2.6 Statistical analysis

Group-wise comparisons for neuropsychological, neuroimaging and biochemical metrics, at each time point, were conducted using an Analysis of Covariance (ANCOVA) after including Age, Sex, APOE4 status and Years of Education as covariates. All analyses were conducted in Python 3.9 using the SciPy and Pingouin packages in Jupyter notebooks.

Survival analysis was conducted using a Cox Proportional Hazards (CPH) model to evaluate the impact of hearing loss on the risk of dementia conversion while adjusting for potential confounders. The model controlled for age, gender, years of education, and APOE4 genotype. The time-to-event outcome was defined as the time (in months) from baseline to dementia conversion or the last available follow-up, whichever occurred first. Survival curves adjusted for the aforementioned covariates were generated for individuals with and without hearing loss.

All analyses were conducted using the lifelines package in Python. A significance level of p < 0.05 was used for all statistical tests.

## 3 Results

### 3.1 Baseline characteristics

**Fig. 3:**
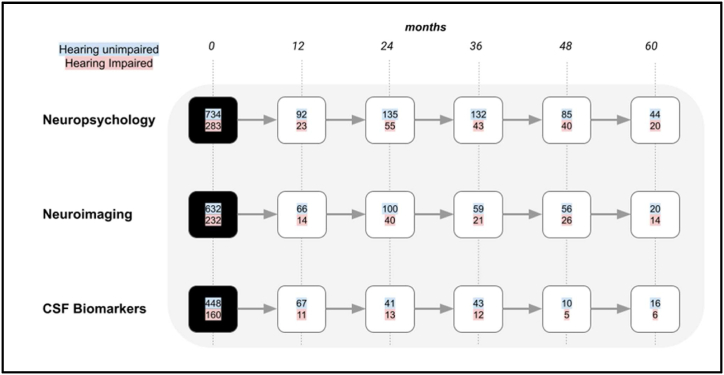
A flowchart showing the number of participants with MCI that converted to a diagnosis of AD dementia at 12-month intervals. The different rows indicate the total number of participants that completed neuropsychological, neuroimaging or CSF biomarker evaluation. The number of hearing unimpaired participants are indicated with a blue background and those with self-reported hearing impairment have a red background. (For interpretation of the references to colour in this figure legend, the reader is referred to the Web version of this article.)

There were 2420 participants in the ADNI study in total with a mean age of 73 years and a standard deviation of 7 years of age at baseline. 411 participants with a diagnosis of AD dementia were excluded from the first part of the study as we were interested in the predictive value of subjective hearing loss for alterations in neuropsychological, neuroimaging or biochemical markers relevant to AD dementia risk. This left 1818 participants in total. Further details regarding baseline characteristics are shown in Table 1. Fig. 3 shows the number of MCI participants who converted to AD dementia at each timepoint which was used to assess the factors affecting dementia risk in this population. This included 669 people who converted to AD within 5 years.

**Table 1:** **-** Baseline participant characteristics.

	Cognitively Normal		Mild Cognitive Impairment	
	Hearing Normal	Hearing Impaired	Hearing Normal	Hearing Impaired
**Count (n)**	605	197	734	283
**Demographics**				
**Age (years)**	73.2 (5.5)	75.9 (5.8)	71.2 (7.3)	75.3 (6.4)
**Gender (n)**				
**Male**	248	116	394	211
**Female**	357	82	340	72
**Education (years)**	16.2 (2.6)	16.9 (2.6)	15.9 (2.9)	16.1 (3.0)
**Cognitive Scores**				
**MMSE (/30)**	29.1 (1.0)	29.1 (1.2)	27.8 (1.8)	27.4 (1.7)
**RAVLT (/50)**	45.6 (9.9)	44.8 (10.0)	34.7 (10.8)	33.0 (9.8)
**Neuroimaging**				
**TIV (ml)**	1496.1 (158)	1538.3 (158)	1545.5 (164)	1556.1 (169)
**Hippocampal volume (ml)**	7.4 (0.9)	7.4 (0.8)	7.0 (1.1)	6.8 (1.1)
**Fluid Biomarkers**				
**pTau-181 +ve**	4	2	20	11

Baseline characteristics for all participants without a diagnosis of AD dementia. MMSE- Mini-Mental State Examination, RAVLT- Rey Adult Verbal Learning Test, TIV- Total Intracranial Volume, pTau-181- Phosphorylated Tau-181. Variability for numerical group level statistics, where applicable, is indicated as a standard deviation in parentheses next to the mean value.

### 3.2 Hearing characteristics

697 people (29 % of total participants) with hearing impairment were identified from participant records. Fig. 1, Fig. 2 show the progression of cognitively normal participants and MCI participants, divided into hearing-unimpaired and hearing-impaired groups, over a five-year period at 12-month intervals. Fig. 3 shows participants with MCI that converted to AD dementia at 12-month intervals up to a follow-up period of five years.

### 3.3 Neuropsychological comparisons

There were no significant differences in MMSE scores between cognitively normal hearing unimpaired and hearing impaired participants at 12 months (*F* (1, 468) = 0.48, *p* = 0.45, np^2^ = 0.001), 24 months (*F* (1, 562) = 3.23, *p* = 0.07, np^2^ = 0.006), 36 months (*F* (1, 240) = 0.85, *p* = 0.36, np^2^ = 0.004), 48 months (*F* (1, 330) = 0.69, *p* = 0.41, np^2^ = 0.002) and 60 months (*F* (1, 130) = 0.01, *p* = 0.91, np^2^ < 0.001), after controlling for age, gender, APOE4 status and years of education.

There were no significant differences in RAVLT scores between cognitively normal hearing unimpaired and hearing impaired participants at 12 months (*F* (1, 466) = 9.72, *p* = 0.002, np^2^ = 0.020), 24 months (*F* (1, 558) = 1.81, *p* = 0.18, np^2^ = 0.003), 36 months (*F* (1, 234) = 1.08, *p* = 0.30, np^2^ = 0.005), 48 months (*F* (1, 327) = 2.81, *p* = 0.09, np^2^ = 0.008) and 60 months (*F* (1, 129) = 1.37, *p* = 0.24, np^2^ = 0.010), after controlling for age, gender, APOE4 status and years of education.

There were no significant differences in MMSE scores between MCI hearing unimpaired and hearing impaired participants at 12 months (*F* (1, 890) = 1.54, *p* = 0.21, np^2^ = 0.002), 24 months (*F* (1, 727) = 0.10, *p* = 0.75, np^2^ < 0.001), 36 months (*F* (1, 612) = 1.21, *p* = 0.27, np^2^ = 0.002), 48 months (*F* (1, 411) = 0.82, *p* = 0.37, np^2^ = 0.002) and 60 months (*F* (1, 239) = 0.44, *p* = 0.51, np^2^ = 0.002), after controlling for age, gender, APOE4 status and years of education.

There were no significant differences in RAVLT scores between MCI hearing unimpaired and hearing impaired participants at 12 months (*F* (1, 887) = 0.09, *p* = 0.77, np^2^ < 0.001), 24 months (*F* (1, 721) = 0.50, *p* = 0.48, np^2^ = 0.001), 36 months (*F* (1, 607) = 0.02, *p* = 0.89, np^2^ < 0.001), 48 months (*F* (1, 404) = 0.30, *p* = 0.58, np^2^ < 0.001) and 60 months (*F* (1, 226) = 0.11, *p* = 0.74, np^2^ = 0.001), after controlling for age, gender, APOE4 status and years of education.

Amongst participants who converted to AD dementia, there were no significant differences in MMSE scores at baseline between hearing unimpaired and hearing impaired participants for those who converted at 12 months (*F* (1, 108) = 0.05, *p* = 0.821, np^2^ < 0.001), 24 months (*F* (1, 187) = 0.41, *p* = 0.52, np^2^ = 0.002), 36 months (*F* (1, 177) = 0.42, *p* = 0.52, np^2^ = 0.002), 48 months (*F* (1, 127) = 0.56, *p* = 0.46, np^2^ = 0.004) and 60 months (*F* (1, 70) = 0.07, *p* = 0.80, np^2^ < 0.001), after controlling for age, gender, APOE4 status and years of education.

Amongst participants who converted to AD dementia, there were no significant differences in RAVLT scores at baseline between hearing unimpaired and hearing impaired participants for those who converted at 12 months (*F* (1, 108) = 0.013, *p* = 0.72, np^2^ = 0.001), 24 months (*F* (1, 187) = 0.33, *p* = 0.57, np^2^ = 0.002), 36 months (*F* (1, 177) = 2.09, *p* = 0.15, np^2^ = 0.012), 48 months (*F* (1, 127) = 1.26, *p* = 0.26, np^2^ = 0.010) and 60 months (*F* (1, 70) = 0.26, *p* = 0.61, np^2^ = 0.004), after controlling for age, gender, APOE4 status and years of education.

### 3.4 Neuroimaging comparisons

There were no significant differences in normalised whole brain volumes between cognitively normal hearing unimpaired and hearing impaired participants at 12 months (F (1, 423) = 0.02, p = 0.89, np^2^ < 0.001), 24 months (F (1, 460) = 0.59, p = 0.44, np^2^ = 0.001), 36 months (F (1, 165) = 0.45, p = 0.50, np^2^ = 0.003), 48 months (F (1, 210) = 3.24, p = 0.07, np^2^ = 0.015) and 60 months (F (1, 95) = 0.04, p = 0.83, np^2^ < 0.001), after controlling for age, gender, APOE4 status and years of education.

There were no significant differences in normalised hippocampal volumes between cognitively normal hearing unimpaired and hearing impaired participants at 12 months (F (1, 377) = 0.01, p = 0.92, np^2^ < 0.001), 24 months (F (1, 425) = 1.23, p = 0.27, np^2^ = 0.003), 36 months (F (1, 194) = 0.12, p = 0.73, np^2^ = 0.001), 48 months (F (1) = 2.81, p = 0.09, np^2^ = 0.008) and 60 months (F (1, 73) = 5.49, p = 0.02, np^2^ = 0.070), after controlling for age, gender, APOE4 status and years of education.

There were no significant differences in normalised whole brain volumes for MCI hearing unimpaired and hearing impaired participants for those who converted at 12 months (F (1, 108) = 0.05, p = 0.821, np^2^ < 0.001), 24 months (F (1, 187) = 0.41, p = 0.52, np^2^ = 0.002), 36 months (F (1, 177) = 0.42, p = 0.52, np^2^ = 0.002), 48 months (F (1, 127) = 0.56, p = 0.46, np^2^ = 0.004) and 60 months (F (1, 70) = 0.07, p = 0.80, np^2^ < 0.001), after controlling for age, gender, APOE4 status and years of education.

There were no significant differences in normalised hippocampal volumes at baseline between MCI hearing unimpaired and hearing impaired participants for those who converted at 12 months (F (1, 634) = 1.41, p = 0.23, np^2^ = 0.002), 24 months (F (1, 505) = 0.05, p = 0.82, np^2^ < 0.001), 36 months (F (1, 227) = 2.22, p = 0.14, np^2^ = 0.010), 48 months (F (1, 229) = 0.04, p = 0.85, np^2^ < 0.001) and 60 months (F (1, 95) = 0.63, p = 0.43, np^2^ = 0.007), after controlling for age, gender, APOE4 status and years of education).

Amongst participants who converted to AD dementia, there were no significant differences in normalised whole brain volumes at baseline between MCI hearing unimpaired and hearing impaired participants at 12 months (F (1, 95) = 0.03, p = 0.86, np^2^ < 0.001), 24 months (F (1, 150) = 0.46, p = 0.50, np^2^ = 0.003), 36 months (F (1, 92) = 0.06, p = 0.80, np^2^ = 0.001), 48 months (F (1, 84) = 0.72, p = 0.40, np^2^ = 0.009) and 60 months (F (1, 33) = 0.29, p = 0.59, np^2^ = 0.009), after controlling for age, gender, APOE4 status.

Amongst participants who converted to AD dementia, there were no significant differences in normalised hippocampal volumes at baseline between cognitively normal hearing unimpaired and hearing impaired participants at 12 months (F (1, 68) = 0.16, p = 0.69, np^2^ = 0.002), 24 months (F (1, 122) = 1.58, p = 0.21, np^2^ = 0.013), 36 months (F (1, 63) = 0.53, p = 0.47, np^2^ = 0.008), 48 months (F (1, 69) = 1.21, p = 0.27, np^2^ = 0.017) and 60 months (F (1, 26) = 1.47, p = 0.24, np^2^ = 0.053), after controlling for age, gender, APOE4 status and years of education.

### 3.5 pTau positivity

There were no significant differences in pTau positivity between cognitively normal hearing unimpaired and hearing impaired participants at 12 months (*F* (1, 87) = 0.07, *p* = 0.80, np^2^ = 0.001), 24 months (*F* (1, 151) = 1.25, *p* = 0.27, np^2^ = 0.008), 36 months (*F* (1, 25) = 1.59, *p* = 0.22, np^2^ = 0.060), 48 months (*F* (1, 64) = 0.25, *p* = 0.62, np^2^ = 0.004) and 60 months (*F* (1, 15) = 1.45, *p* = 0.25, np^2^ = 0.089), after controlling for age, gender, APOE4 status and years of education and Bonferroni correction for multiple comparisons.

There were no significant differences in pTau positivity between MCI hearing unimpaired and hearing impaired participants at 12 months (*F* (1, 148) = 1.34, *p* = 0.25, np^2^ = 0.009), 24 months (*F* (1, 246) = 0.55, *p* = 0.46, np^2^ = 0.002), 36 months (*F* (1, 38) = 4.18, *p* = 0.05, np^2^ = 0.010), 48 months (*F* (1, 104) = 0.34, *p* = 0.56, np^2^ = 0.003) and 60 months (*F* (1, 16) = 1.22, *p* = 0.28, np^2^ = 0.071), after controlling for age, gender, APOE4 status and years of education and Bonferroni correction for multiple comparisons.

Amongst participants who converted to AD dementia, there were no significant differences in pTau positivity at baseline between hearing unimpaired and hearing impaired participants for those who converted at 12 months (*F* (1, 72) = 0.35, *p* = 0.55, np^2^ = 0.005), 24 months (*F* (1, 48) = 3.60, *p* = 0.06, np^2^ = 0.070), 36 months (*F* (1, 49) = 0.24, *p* = 0.63, np^2^ = 0.005), 48 months (*F* (1, 9) = 0.54, *p* = 0.48, np^2^ = 0.057) and 60 months (*F* (1, 16) = 0.08, *p* = 0.78, np^2^ = 0.004), after controlling for age, gender, APOE4 status and years of education and Bonferroni correction for multiple comparisons.

### 3.6 Survival analysis of hearing loss as a risk factor for AD dementia

**Fig. 4:**
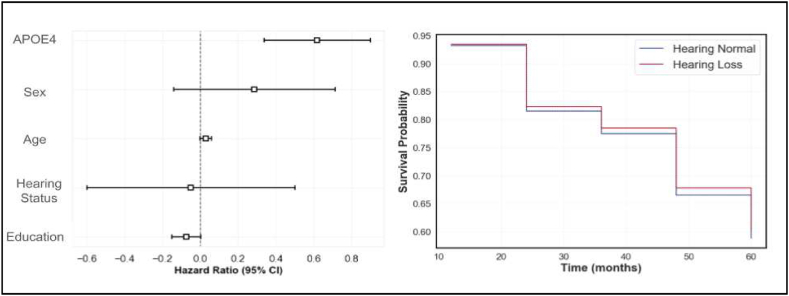
Cox Proportional Hazard (CPH) analysis for conversion from MCI to AD Dementia. The image on the left shows the hazard ratios estimated by the CPH model for various covariates. APOE4 status has the largest impact on conversion to dementia, followed by age. The image on the right shows adjusted survival curves for people with (HL) (blue line) and without (HN) (red line) hearing loss. There is no significant impact on dementia conversion based on hearing status. CI – Confidence Interval. (For interpretation of the references to colour in this figure legend, the reader is referred to the Web version of this article.)

The CPH model was used to assess the risk of dementia conversion in relation to hearing loss while adjusting for age, gender, years of education, and APOE4 genotype. The adjusted survival curves for individuals with and without hearing loss overlapped considerably throughout the observation period, suggesting similar survival probabilities between the two groups. This is indicated by the coefficients of the model in Table 2 and Fig. 4.

**Table 2:** **-** Cox proportional hazard model summary.

	Coefficient	z-score	p-value
Age	0.03 (0.02–0.06)*	2.02	0.04
Sex	0.29 (−0.14 to 0.71)	1.31	0.19
Education	−0.07 (−0.15 to 0.00)	−1.88	0.06
APOE4	0.66 (0.50–0.81)*	4.32	<0.005
Hearing Loss	−0.05 (−0.60 to 0.50)	−0.17	0.86

Results of the Cox Proportional Hazard model for predicting conversion from MCI to AD dementia in the ADNI cohort. Separate covariate parameter coefficients, the z-scores associated with these and their p-values are shown. Confidence intervals for the coefficients are shown in parenthesis. Asterisks indicate statistically significant values.

Specifically, subjective hearing loss status was not found to be a significant predictor of dementia conversion. The hazard ratio for hearing loss, compared to no hearing loss, was −0.05 (95 % CI: -0.60 to 0.50, p = 0.86). This indicates that individuals with hearing loss did not have a significantly different risk of converting to dementia compared to those without hearing loss, after adjusting for the included covariates.

## 4 Discussion

This study was designed to assess whether the presence of subjective hearing loss is predictive of AD dementia through changes in clinically relevant cognitive, neuroimaging or cerebrospinal fluid biochemical markers of AD. We found that there were no significant differences between hearing impaired and hearing unimpaired participants in cognitively normal and people with MCI over a 5-year period nor were there any group-level changes in these variables in people who had converted from MCI to AD dementia. Subjective hearing impairment may thus have a limited value in predicting AD dementia risk.

Episodic memory impairment is a hallmark of AD dementia and forms part of the initial diagnostic criteria for the condition [22]. Decline in episodic memory on the RAVLT and other tests of delayed verbal recall has been shown to predict AD dementia development [23,24]. Subjective hearing loss has also been linked to an increased risk of cognitive impairment compared to hearing unimpaired individuals [25]. A decline in episodic memory scores in preclinical AD, however, has not been found to be predictive of AD itself in other studies [26]. In this study, we did not find a faster decline in RAVLT scores in people with subjective hearing impairment in cognitively unimpaired individuals or those with MCI, over a 5-year period. This would indicate that there may not be early cognitive changes relevant to the risk for AD dementia in those with subjective hearing loss. Participants with MCI who converted to AD dementia over a 5-year period also did not have faster cognitive decline than hearing unimpaired participants. These findings suggest that subjective hearing impairment may not be directly related to early cognitive markers of AD dementia over a shorter time span.

The most common form of AD dementia is also associated with hippocampal volume loss and atrophy in this region can also increase the risk of AD dementia in healthy individuals and those with pre-clinical AD [27,28]. Hearing loss, as defined by pure tone audiometry, has also been associated with faster rates of medial temporal lobe and hippocampal atrophy [6,29]. However, our study did not find any group-level differences between participants with subjective hearing impairment and hearing unimpaired participants after 5-years. Participants with mild cognitive impairment who converted to AD dementia over this time period also did not have any brain metric differences at baseline that would help predict dementia. Therefore, subjective hearing impairment may not lead to greater hippocampal atrophy in these populations over time.

There is great interest in neurodegenerative markers, which can predict conversion to AD dementia with a high degree of accuracy [30]. Phosphorylated tau-181 is predictive of conversion and we used a potentially clinically relevant cut-off [21]. Previous studies have found an association between subjective hearing loss and raised pTau-181 in the ADNI cohort [19]. However, it is unclear whether this actually increases the risk of AD dementia in the future. We did not replicate this finding with the most updated version of the ADNI dataset. Neuropathological changes are also more evident in hearing impaired individuals compared to hearing unimpaired individuals [11]. However, we did not find that pTau-181 was preferentially predictive of AD dementia conversion over 5 years in the participants with subjective hearing loss.

The longitudinal study design and the use of clinically relevant predictive markers of AD diagnosis as outcome variables is a strength of this study. We analysed metrics that could differentiate people at greater risk of AD dementia if subjective hearing loss instantiated this risk. This would allow a quick assessment of subjective hearing loss to be used alongside cognitive, neuroimaging and biochemical testing in the clinical environment. The study was conducted with a comprehensive dataset from the ADNI study and with participants where hearing loss is most likely to have an influence on disease markers, if a true neurobiological link between subjective hearing loss and dementia exists. However, subjective hearing loss was not associated with group level differences in the chosen variables.

There are possible explanations for lack of association between subjective hearing loss and established markers that predict AD dementia. Firstly, there is a poor association between subjective and objective hearing measures which may not capture the evidence link between hearing loss and subsequent dementia [31]. One study found the use of subjective hearing impairment over represents the presence of hearing loss as over half of participants did not have hearing loss as defined by thresholds [32]. Although subjective hearing loss correlates with hearing disability, it is insensitive to age effects. It overestimates hearing loss below 70 years of age and underestimates that in people above 70 years of age [33]. Therefore, it is possible the specific subjective hearing loss metric used in this study may not capture the relationship that has been identified between objective hearing loss and future dementia risk and a more comprehensive one is required. Other mechanisms that are not related to the neuropathological hallmarks of AD dementia also need to be studied further. It is likely that there are multiple factors that influence an individual's decision to report if they feel they have hearing loss such as the activities they carry out and the environments these occur in, mental health, stigma about hearing loss or how self-aware an individual may be about any hearing difficulties.

Another factor may have been that the percentage of hearing impaired participants was around 30 % in our cohort at a mean age of 73 years of age when one would expect greater than 70 % to have some form of hearing loss [2]. The lack of objective data on hearing in ADNI made it difficult to establish whether milder hearing loss was overrepresented in our cohort where one would expect a weaker link between hearing loss and dementia risk. Subjective hearing loss may also be more prevalent than objective hearing loss determined by pure-tone audiometry and this overrepresentation may then result in people included in a cohort with ‘hearing loss’ that may dilute any effects of objective hearing loss and risk of dementia. Data on when an individual acquired hearing loss as it may be associated with a greater risk of dementia at early rather than later stages [3]. Finally, in this study, people with hearing impairment were less likely to complete the follow-up period of 5 years and had a bigger drop-off at each time point. The reasons for this are unclear. This censoring effect may have contributed to the lack of identification of an association between subjective hearing loss and AD dementia risk.

The subjective nature of the hearing loss data means that some of the responses that were collected may have been influenced by interventions such as hearing aids. For example a person who may have had hearing loss subjectively may have felt that their hearing impairment had improved to the extent that they were not troubled by it or that the fact that they were using hearing aids meant that their difficulties were ‘addressed’. The fact that someone may have been a hearing-aid user may not have influenced the results dramatically as compliance and usage patterns vary from individual to individual (J et al., 2013). However, there may have been cognitive benefits to certain populations, such as those with cardiovascular risk factors who have been shown to potentially benefit from their use and so our findings should be taken with these findings in mind [34].

It is possible that subjective hearing loss increases a person's future AD dementia risk by other mechanisms. Further work is necessary to assess whether subjective hearing ability is related to an individual's resilience against dementia and then be a useful metric in that regard. Sensory loss, in general, may give an indication of an individual's resilience against dementia [35]. Metacognitive markers like subjective memory loss have been shown to have a similar risk for dementia as early mild cognitive impairment but the mechanisms for this relationship are unclear [40]. It is also possible that neuropathology related to other dementia syndromes, rather than AD, is better associated with hearing loss. Poor subjective hearing is also associated with poor quality of life and there may be psychological factors such as low mood at play which independently predict dementia risk [36,37]. Self reported hearing handicap, rather than clinical measured hearing impairment, predicted a decline in quality of life over a 10-year period suggesting that associated psychological and social factors may be more important in determining well-being in older adults.

## 5 Conclusions

The findings from our study have implications for the analysis of dementia risk from other large-scale cohorts. Information regarding hearing status is usually captured in a dichotomous form, as was performed in this study, based on the presence or absence of self-reported hearing difficulties. Despite being a quick measurement of hearing status, it may not be able to replace objective hearing measurements of pure-tone audiometry thresholds or speech-in-noise perception ability which have been directly associated with future dementia risk. Even if there are clear individual risks for dementia associated with subjective hearing loss these may be due to psychological factors independently associated with the condition. Future cohort studies or amendments to existing cohort studies should include an assessment of objective hearing metrics so that the mechanisms for the association between hearing loss and dementia can be clarified.

## Ethics approval

This study was approved by the Newcastle University Faculty of Medical Sciences Ethics Committee.

## Data availability statement

Data from the manuscript was taken from the ADNI database and is available to registered and approved researchers.

## CRediT authorship contribution statement

**Meher Lad:** Writing – review & editing, Writing – original draft, Visualization, Validation, Software, Resources, Project administration, Methodology, Investigation, Formal analysis, Data curation, Conceptualization. **John-Paul Taylor:** Writing – review & editing. **Tim D. Griffiths:** Writing – review & editing.

## Declaration of competing interest

The authors declare that they have no known competing financial interests or personal relationships that could have appeared to influence the work reported in this paper.

## Abbreviations

AD: Alzheimer's disease
MCI: Mild Cognitive Impairment
RAVLT: Rey Auditory Verbal Learning Test

[38,39].
